# Acute Arteritis. La Pitié

**Published:** 1831

**Authors:** 


### XLIX.

### Acute Arteritis. La Pitié.

M. Camus reports the following case from the wards of La Pitié.

*Case.* Charles Tava, a negro, aged 28 years, entered the hospital on the 30th December, 1830, for a painful engorgement of the left testicle. Leeches, poultices, fomentations, removed the swelling, and the man appeared to be nearly fit for discharge, when, all at once, he complained of great difficulty of breathing, spitting of blood, and extreme frequency of pulse. Auscultation did not throw any light on the symptoms. Diluents were prescribed. Next day, his condition was still worse. On the fourth day he was menaced with suffocation, and the radial artery was *not perceptible*. And now for the first time the patient was ordered "une tres petit saignée explorative," and the poor negro expired a few minutes afterwards.

*Dissection.* The lungs were gorged with blood of a very thin kind, but were every where pervious to air. There were no tubercles. The internal surface of the chambers of the heart was of a livid red colour, especially about the valves of the aorta. This redness extended some way into the substance of the parietes; and also through the aorta and pulmonary arteries, to the iliac bifurcation of the former, and to the capillary extremities of the latter. The internal membrane of the vessels could be easily detached, leaving the subjacent tissues tinged of a red colour. The iliacs and femoral arteries presented a grade of the same tinge. The same might be said of the carotids and subclavians. The cava inferior also shewed a slight appearance of the same kind.

The pathologist will balance between the conflicting opinions of arteritis and imbibition post-mortem. We forgot to mention that the dissection, in the present case, took place 40 hours after death, and consequently after sufficient time had been given for the colouring matters of the blood to act forcibly on the vessels containing that fluid. Still it must be recollected the extraordinary violence of the symptoms could not be accounted for by any of the post-mortem appearances, if we except the apparent inflammation of the vessels.

